# Gastric residual volume, safety, and effectiveness of drinking 250 mL of glucose solution 2–3 hours before surgery in gastric cancer patients: a multicenter, single-blind, randomized–controlled trial

**DOI:** 10.1093/gastro/goae077

**Published:** 2024-09-12

**Authors:** Dongjie Yang, Xun Hou, Huafeng Fu, Wu Song, Wenqing Dong, Hu Wang, Yuantian Mao, Mengbin Li, Junqiang Chen, Yulong He

**Affiliations:** Digestive Medicine Center, The Seventh Affiliated Hospital, Sun Yat-sen University, Shenzhen, Guangdong, P. R. China; Guangdong Provincial Key Laboratory of Digestive Cancer Research, Shenzhen, Guangdong, P. R. China; Research Center for Diagnosis and Treatment of Gastric Cancer, Sun Yat-sen University, Guangzhou, Guangdong, P. R. China; Center for Gastrointestinal Surgery, The First Affiliated Hospital, Sun Yat-sen University, Guangzhou, Guangdong, P. R. China; Digestive Medicine Center, The Seventh Affiliated Hospital, Sun Yat-sen University, Shenzhen, Guangdong, P. R. China; Center for Gastrointestinal Surgery, The First Affiliated Hospital, Sun Yat-sen University, Guangzhou, Guangdong, P. R. China; Center for Gastrointestinal Surgery, The First Affiliated Hospital, Sun Yat-sen University, Guangzhou, Guangdong, P. R. China; Department of Gastrointestinal Surgery, The Forth Military Medical University Xijing Hospital, Xi’an, Shaanxi, P. R. China; Department of Gastrointestinal Surgery, The First Affiliated Hospital of Guangxi Medical University, Nanning, Guangxi, P. R. China; Department of Gastrointestinal Surgery, The Forth Military Medical University Xijing Hospital, Xi’an, Shaanxi, P. R. China; Department of Gastrointestinal Surgery, The First Affiliated Hospital of Guangxi Medical University, Nanning, Guangxi, P. R. China; Digestive Medicine Center, The Seventh Affiliated Hospital, Sun Yat-sen University, Shenzhen, Guangdong, P. R. China; Guangdong Provincial Key Laboratory of Digestive Cancer Research, Shenzhen, Guangdong, P. R. China; Research Center for Diagnosis and Treatment of Gastric Cancer, Sun Yat-sen University, Guangzhou, Guangdong, P. R. China; Center for Gastrointestinal Surgery, The First Affiliated Hospital, Sun Yat-sen University, Guangzhou, Guangdong, P. R. China

**Keywords:** gastric cancer, preoperative drinking, 5%, glucose solution, gastric residue volume, perioperative complications

## Abstract

**Background:**

Carbohydrate drinking 2–3 hours before surgery has been widely adopted in colorectal operations. However, there is little direct evidence regarding its application in gastric cancer surgery. We aimed to evaluate the gastric residual volume, safety, and effectiveness of drinking 250 mL of 5% glucose solution 2–3 hours before elective gastric cancer surgery.

**Methods:**

We conducted an investigator-initiated, multicenter, randomized–controlled, parallel group, and equivalence trial. Eighty-eight patients with gastric adenocarcinoma were randomized into study or control group. Patients in the control group followed the traditional routine of 6–8 hours preoperative fasting, while those in the study group drank 250 mL of 5% glucose solution 2–3 hours before surgery. Immediately following tracheal intubation, gastric contents were aspirated through gastroscopy. The primary outcome was preoperative gastric residual volume.

**Results:**

Eighty-three patients were eventually analysed in the study (42 in the study group and 41 in the control group). Two groups were comparable at baseline characteristics. There were no statistical differences in residual gastric fluid volumes (35.86 ± 27.13 vs 27.70 ± 20.37 mL, *P *=* *0.135) and pH values (2.81 ± 1.99 vs 2.66 ± 1.68, *P *=* *0.708) between the two groups. Preoperative discomfort was significantly more decreased in the study group than in the control group (thirst score: 1.49 ± 1.23 vs 4.14 ± 2.07, *P *<* *0.001; hunger score: 1.66 ± 1.18 vs 3.00 ± 2.32, *P *=* *0.007). There was no statistical difference in the incidence of postoperative complications (19.05% vs 17.07%, *P *=* *0.815).

**Conclusions:**

Drinking 250 mL of 5% glucose solution 2–3 hours before surgery in elective gastric cancer patients shows benefits in lowering thirst and hunger scores without increasing gastric residual volume and perioperative complications.

## Introduction

For decades, overnight fasting has been routine in elective surgery to reduce gastric residual volume before anesthesia, which in turn reduces the risk of perioperative aspiration pneumonia [1]. However, in clinical practice, the observed incidence of perioperative aspiration is relatively low [2], while prolonged fasting might cause adverse reactions such as headache, dehydration, hypovolemia, and hypoglycemia, etc. [3]. In 1997, Kehlet reported their experience of “multimodal rehabilitation”, which was later known as “fast-track surgery” or “enhanced recovery after surgery (ERAS)” [4]. Many studies thereafter confirmed the results from Kehlet, including our own report on colorectal cancer surgery [5]. Nowadays, reduced preoperative fasting time and preoperative oral intake of carbohydrate solution have been advocated in elective colorectal cancer surgery [6].

Gastric cancer is the fifth most commonly diagnosed cancer and the third leading cause of cancer death worldwide [7]. In China, it ranks at second in terms of incidence and mortality, accounting for 456,124 new cases and 390,182 deaths annually [8]. Radical gastrectomy under general anesthesia is the standard surgical treatment for gastric cancer, with overnight fasting still being routine practice. Although several studies in China, Korea, and Japan have demonstrated that ERAS protocols including shortened fasting time and preoperative carbohydrate drinking could be beneficial for patients with gastric cancer [9–11], none of those offered direct observation of the gastric residue volume and pH value, not to mention the other ERAS elements that might obscure the true effect of reduced fasting time and preoperative carbohydrate drinking alone. The present trial was designed to explore the impact of preoperative carbohydrate drinking on gastric emptying and the pH of gastric fluid for the first time through gastroscopy, and to evaluate perioperative outcomes in gastric cancer patients and thereby provide direct evidence for clinical practice in the future.

## Methods

### Study design and participants

This was an investigator-initiated, multicenter, randomized–controlled, parallel group, equivalence trial to evaluate the safety and effectiveness of administrating preoperative oral carbohydrate solution in elective gastric cancer surgery (clinicaltrials.gov, NCT02815150). We conducted the study with the approval of the Research Ethics Committee of the First Affiliated Hospital, Sun Yat-sen University (Guangzhou, P. R. China), the First Affiliated Hospital of Guangxi Medical University (Nanning, P. R. China), and the Forth Military Medical University Xijing Hospital (Xi’an, P. R. China). Written informed consent was obtained from all patients before enrollment.

Inclusion criteria were as follows: (i) age between 18 and 80 years; (ii) histologically confirmed gastric adenocarcinoma; (iii) tumor of cT2–4aN0–2 on preoperative gastroscopy, endoscopic ultrasound, and/or abdominal computed tomography, in which tumor T, N, M staging was performed according to the tumor, node, metastasis (TNM) staging criteria of the 7th edition of the American Joint Committee on Cancer (AJCC) [12]; (iv) fit for elective radical resection; (v) Eastern Cooperative Oncology Group performance status of 0 or 1; (vi) American Society of Anesthesiology (ASA) status of I–III; (vii) body mass index (BMI) of 17.5–27.5 kg/m^2^; and (viii) patient agreed to participate in this trial through informed consent.

Patients with (i) symptoms of pyloric obstruction; (ii) impaired bowel function, using drugs disturbing gastric secretion and gastric emptying; (iii) history of gastric resection; (iv) history of treatment for gastric cancer; (v) history of major abdominal operation, or history of diffuse peritonitis; (vi) presence of diabetes, impaired glucose tolerance, or other endocrine hormone abnormalities; (vii) potential difficult airway as evaluated by anesthesiologist; or (viii) pregnancy or breastfeeding were excluded in this trial. Patients were also excluded if they had: (i) failure of endotracheal intubation, (ii) failure of intraoperative gastroscopy, or (iii) unresectable tumor.

After initial screening, eligible patients were randomized in a 1:1 ratio to control and study groups. In order to minimize the influence of time-related confounding factors such as season while ensuring similar progress in both groups, the total 88 cases were divided into 11 segments equally and all cases were randomized within segments. The random number generator of Microsoft Office Excel 2011 was adopted to generate eight random numbers for each segment to create a randomized code table. Surgeons, anesthetists, doctors performing endoscope, co-workers collecting specimens, follow-up visitors, and statisticians were uninformed of the randomization.

### Intervention

The study intervention was drinking 250 mL of 5% glucose solution (China Otsuka Pharmaceutical, Beijing, P. R. China). All oral solutions were kept at 37°C until being drunk. Patients in the study group drank the solution 2–3 hours before surgery. Patients in the control group followed the traditional routine of preoperative fasting for 6–8 hours.

### Perioperative management

Bowel preparation was not routinely done before surgery. The prophylactic antibiotic was administered half an hour before the induction of anesthesia and once more after 3 hours. We adhered to the Japanese Guidelines for Gastric Cancer Treatment (4th edition) for the surgical approach, extent of gastrectomy, lymph node dissection, and method of gastro-intestinal reconstruction [13]. A majority of patients received minimally invasive surgery, including laparoscopic and robotic surgery. A nasogastric tube was not routinely placed after surgery and only if the risk of anastomotic leakage or bleeding was high (e.g. severe gastric wall edema, dissatisfied anastomosis, etc.). Two abdominal drainage tubes were placed routinely. The tip of the left drainage tube was positioned in the splenic fossa. The right drainage tube was laid beneath the left lobe of the liver, with its proximal end passing through the Winslow's foramen and positioned 3 cm from the gastroesophageal junction. The time to remove the nasogastric tube and abdominal drainage tube was determined by surgeons according to postoperative recovery. The urinary catheter was removed when patients were conscious and could mobilize out of bed. A combination of thoracic epidural local anesthetics plus non-specific anti-inflammatory drugs was used to control postoperative pain. The time to start a liquid diet was decided by the surgeons. As routine practice, all patients had well-fitting compression stockings on and were encouraged to mobilize after surgery. All patients were given parenteral nutrition from the first postoperative day. Discharge criteria were as follows: normal body temperature; independently mobile; return of normal gastro-intestinal function (defecation at least once); normal oral diet, no need for parenteral nutrition; controllable pain with oral analgesia; willing to go home.

### Outcomes

The primary outcome of this study was preoperative gastric residual volume. General anesthesia was induced with 100–120 mg of propofol and 0.5 mg/kg of rocuronium bromide, followed by tracheal intubation. A gastroscope (Olympus 260, Japan) was then inserted and complete suction of gastric fluid was performed under direct vision, with the suction apparatus connected to a sterile airtight collector. The volume of gastric residual fluid in the collector was measured by using a cylindrical measuring glass.

The secondary end-points included pH of the gastric residual fluid, preoperative thirst and hunger status, perioperative insulin sensitivity, early surgical outcomes, recovery of bowel function, incidence of perioperative complications, length of postoperative hospital stay, and readmission 30 days after surgery.

The pH of the gastric fluid was measured by using a Delta 320 pH Detector (Mettler Toledo, Switzerland).

Preoperative discomfort such as thirst and hunger was evaluated by using a 10-cm Visual Analog Scale, with 0 cm being no thirst or hunger and 10 cm being the worst imaginable thirst and hunger. All scales were reported by patients and collected by a research assistant immediately before anesthesia induction.

Blood glucose and insulin levels were measured at the following time spots: 6 a.m. on the same day before surgery (after overnight fasting) as the baseline data, right after surgery (after skin closure), and 6 a.m. on postoperative days (PODs) 1, 3, and 5 (after overnight fasting). The insulin sensitivity of each time spot was determined by calculating the quantitative insulin sensitivity check index (QUICKI) [14]. By definition, QUICKI = 1/[log (I0) + log (G0)], where I0 is the fasting insulin level and G0 is the fasting glucose level. Recovery of QUICKI was calculated as postoperative QUICKI divided by baseline QUICKI. Perioperative laboratory findings representing infections such as C-reactive protein (CRP) and white blood cell (WBC) levels were also recorded.

Early surgical outcomes such as duration of surgery, intraoperative blood loss, fluid infusion, and urine output were recorded. The time to first oral intake, flatus, and defecation after surgery was observed to evaluate recovery of bowel function.

Postoperative complication was defined as any surgery-related complications occurring within 30 days after surgery. Readmission and surgical mortality were defined as any admission and death due to surgical complications within 30 days after surgery, respectively.

### Sample size

This study was designed as an equivalence trial. The equivalence margins were set at –10.0 and 20.0 mL for the primary outcome, meaning that the lower bound of the two-sided 95% confidence interval (CI) for the difference gastric fluid volumes between the two arms should exceed –10 mL and the upper bound should not exceed 20 mL. According to our previous study, the standard deviation of gastric residual fluid volumes was set at 15.0 mL [15]. Assuming that the true difference in gastric residual fluid volumes between the two arms was the same, a sample size of 88 patients was needed to achieve a power of 90% to establish the equivalence of the two arms at a 5.0% significance level. This calculation allowed for a rate of withdrawal and loss to follow-up of 10%.

### Statistical analysis

Numbers (percentages) were calculated for categorical variables and mean ± standard deviation (SD) or median (interquartile range) for continuous variables. Comparisons between the two groups were performed by using the independent Student’s *t*-test or Mann–Whitney *U* test for continuous variables and the chi-square test or Fisher’s exact test for categorical variables. The repeated measurements were compared by using linear mixture models, with groups as fixed effects and time, and the interaction of time and group as a random effect. All the analyses were conducted using SPSS 25.0 (IBM SPSS Statistics, IBM, Armonk, NY, USA).

## Results

### Patients’ demographics

**Figure 1.**
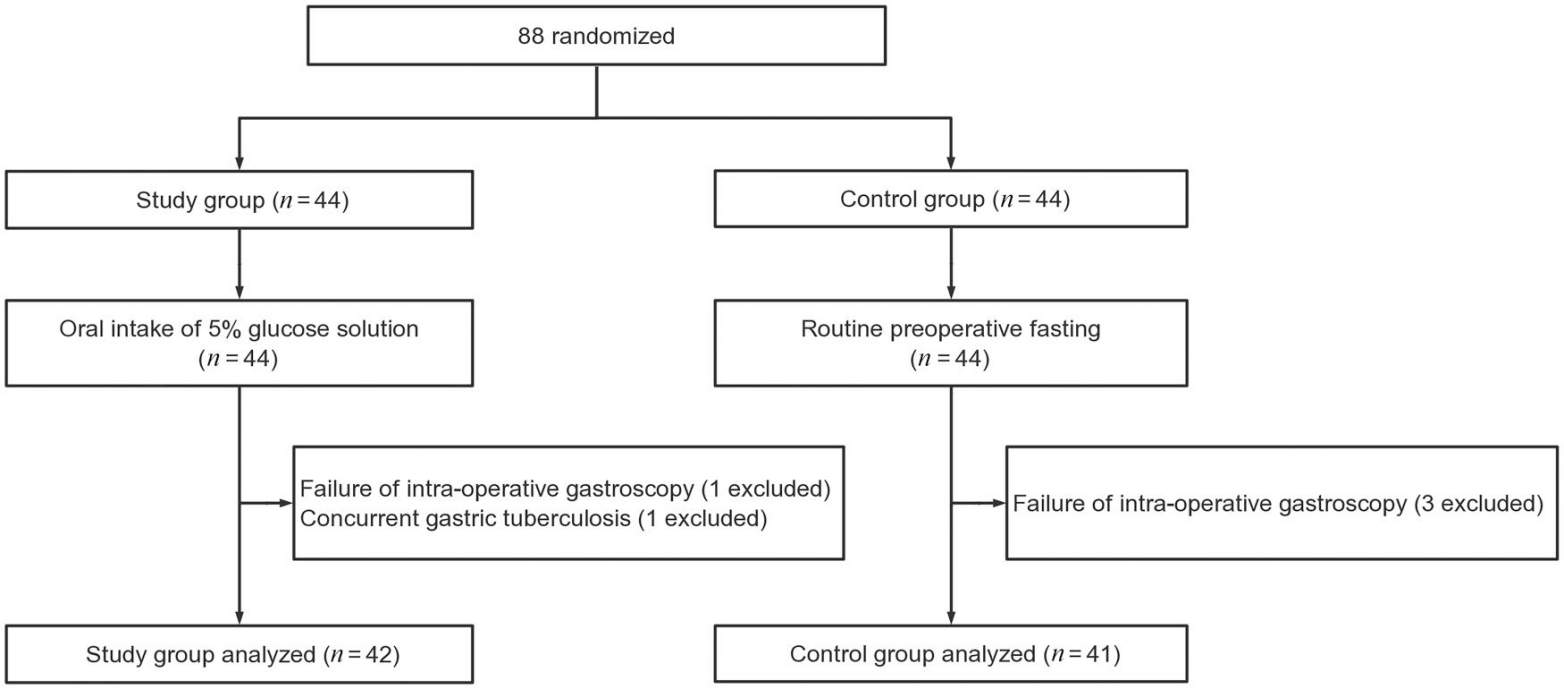
CONSORT flowchart of this study

Figure 1 presents a flow chart of the trial. From April 2016 to December 2018, 88 patients were recruited and randomized into the study group (44 patients) and control group (44 patients). Two patients were excluded from the study group because of intraoperative gastroscopy failure (one patient) and postoperative pathologically confirmed coexisting tuberculosis (one patient), respectively. The reason we excluded the latter patient was that the intra-abdominal adhesion and overall feebleness caused by the tuberculosis might have jeopardized the outcome of the study. Three patients from the control group were excluded because of intraoperative gastroscopy failure. Finally, 42 patients in the study group and 41 in the control group completed the study.

The baseline characteristics of the study and control groups were well balanced. There were no statistical differences in age, sex, BMI, preoperative hemoglobin (Hb), WBC, albumin (ALB), prealbumin (preALB), globulin (GLB), tumor location, tumor staging, types of surgery, resection, and reconstruction between the study and control groups (Table 1).

**Table 1.** Demographic, physiological, and surgical characteristics of patients at baseline

Variables	Study group (*n *=* *42)	Control group (*n *=* *41)	*P-*value
Age, years, mean ± SD	54 ± 16	54 ± 10	0.795
Male, *n* (%)	26 (61.9)	24 (58.5)	0.754
BMI, kg/m^2^, mean ± SD	22.88 ± 4.11	23.31 ± 3.68	0.477
WBC, ×10^9^/L, median (IQR)	5.94 (3.93 − 10.72)	5.92 (3.57 − 10.29)	0.895
Hb, g/L, mean ± SD	136.0 (90 − 179)	141.0 (70 − 169)	0.610
ALB, g/L, mean ± SD	40.2 ± 3.85	40.5 ± 3.39	0.982
preALB, g/L, mean ± SD	235.9 ± 49.7	248.4 ± 45.4	0.294
GLB, g/L, mean ± SD	26.9 ± 3.82	26.1 ± 3.69	0.332
Tumor location, *n* (%)			0.562
Fundus	7 (16.7)	5 (12.2)	
Body/antrum	35 (83.3)	36 (87.8)	
TNM classification, *n* (%)			0.722
0	1 (2.4)	2 (4.9)	
I	16 (38.1)	18 (43.9)	
II	11 (26.2)	7 (17.1)	
III	14 (33.3)	14 (34.1)	
Type of surgery, *n* (%)			0.178
Open	13 (31.0)	6 (14.6)	
Laparoscopic	27 (64.3)	31 (75.6)	
Robotic	2 (4.8)	4 (9.8)	
Type of resection, *n* (%)			0.426
Distal gastrectomy	28 (66.7)	33 (80.5)	
Proximal gastrectomy	2 (4.8)	1 (2.4)	
Total gastrectomy	12 (28.6)	7 (17.1)	
Type of reconstruction, *n* (%)			0.104
Billroth I	0 (0)	1 (2.4)	
Billroth II	24 (57.1)	30 (73.2)	
Roux-en-Y	18 (42.9)	10 (24.4)	

ALB = albumin, BMI = body mass index, GLB = globulin, Hb = hemoglobin, preALB = prealbumin, WBC = white blood cells.

### Gastric residual volume and pH, and preoperative discomfort

As shown in Table 2, there was no significant difference in the gastric residual volume and pH between the study and control groups (35.86 ± 27.13 vs 27.70 ± 20.37 mL, *P *=* *0.135; 2.81 ± 1.99 vs 2.66 ± 1.68, *P *=* *0.708). Preoperative thirst and hunger scores were significantly lower in the study group (thirst score: 1.49 ± 1.23 vs 4.14 ± 2.07, *P *<* *0.001; hunger score: 1.66 ± 1.18 vs 3.00 ± 2.32, *P *=* *0.007).

**Table 2.** Volume and pH of gastric residual and preoperative discomfort

Variables	Study group (*n *=* *42)	Control group (*n *=* *41)	Mean difference (95% CI)	*P*-value
Gastric residual volume, mL	35.86 ± 27.13	27.70 ± 20.37	8.16 (–2.54 to 18.48)	0.135
Gastric residual pH	2.81 ± 1.99	2.66 ± 1.68	0.16 (–0.68 to 0.99)	0.708
Preoperative discomfort				
Thirst	1.49 ± 1.23	4.14 ± 2.07	–2.65 (–3.52 to –1.86)	<0.001
Hunger	1.66 ± 1.18	3.00 ± 2.32	–1.34 (–2.29 to –0.40)	0.007

CI = confidence interval.

### Insulin sensitivity and laboratory findings

Insulin sensitivity is represented as QUICKI in Table 3. At each time point, QUICKI was not significantly different between the two groups (all *P *>* *0.05). Calculation of the recovery of insulin sensitivity revealed that the difference between the two groups was also not significant (all *P *>* *0.05). Regarding laboratory findings representing systemic inflammation such as CRP and WBC levels, there were no significant differences between the two groups in each time spot (Table 3, all *P *>* *0.05). No statistical differences in both status and recovery of QUICKI, WBC, and CRP were noted for group or group×time in linear mixed model analyses (all *P *>* *0.05, Supplementary Figure 1).

**Table 3.** Perioperative insulin sensitivity and laboratory findings

Variables	Study group (*n *=* *42)	Control group (*n *=* *41)	*P-*value
QUICKI, mean ± SD			
Baseline	0.382 ± 0.048	0.392 ± 0.051	0.455
Post-op	0.358 ± 0.037	0.363 ± 0.043	0.635
POD1	0.358 ± 0.052	0.351 ± 0.041	0.552
POD3	0.339 ± 0.039	0.351 ± 0.048	0.264
POD7	0.374 ± 0.047	0.392 ± 0.049	0.266
Recovery of QUICKI, %, mean ± SD			
Post-op	94.2 ± 13.1	91.9 ± 13.9	0.568
POD1	95.7 ± 13.4	90.2 ± 12.6	0.101
POD3	90.0 ± 15.7	89.4 ± 10.0	0.905
POD7	95.0 ± 10.0	99.3 ± 10.8	0.207
WBC, × 10^9^/L, median (IQR)			
Post-op	12.0 (5.18–19.32)	11.7 (5.01–30.21)	0.670
POD1	12.5 (5.83–31.32)	12.3 (5.55–23.71)	0.591
POD3	9.9 (4.12–19.07)	9.4 (3.59–18.56)	0.791
POD7	9.8 (5.42–15.08)	11.2 (4.56–26.34)	0.540
CRP, mg/L, median (IQR)			
Post-op	2.1 (0.5–25.1)	3.0 (0.4–146.4)	0.976
POD1	86.0 (29.2–161.9)	79.3 (1.0–301.2)	0.826
POD3	135.5 (46.4–269.7)	132.8 (18.0–191.8)	0.455
POD7	68.2 (9.0–177.0)	81.5 (19.0–301.0)	0.944

Baseline = the day before surgery, CRP = C-reactive protein, IQR = interquartile range, POD = postoperative day, Post-op = right after operation, SD = standard deviation, QUICKI = quantitative insulin sensitivity check index, WBC = white blood cells.

### Early surgical outcomes and postoperative course

Early surgical outcomes and postoperative recovery results are listed in Table 4. Operation time (286 vs 250 min, *P *=* *0.085), intraoperative blood loss (100 vs 100 mL, *P *=* *0.416), fluid infusion (2,800 vs 2,700 mL, *P *=* *0.577), and urine output (750 vs 600 mL, *P *=* *0.104) were similar between the study and control groups. There were no aspirations in either group.

**Table 4.** Early surgical outcomes and postoperative course of patients (*n *=* *83)

Variables	Study group (*n *=* *42)	Control group (*n *=* *41)	*P-*value
Operation time, min, median (IQR)	286 (140–663)	250 (150–460)	0.085
Intraoperative blood loss, mL, median (IQR)	100 (20–500)	100 (0–850)	0.416
Intraoperative fluid infusion, mL, median (IQR)	2,800 (1,500–5,700)	2,700 (1,500–4,500)	0.577
Intraoperative urine output, mL, median (IQR)	750 (0–2,000)	600 (0–1,550)	0.104
Recovery of bowel function, days, median (IQR)			
Time to first flatus	3 (1–5)	3 (1–9)	0.322
Time to first defecation	4 (2–7)	4 (1–10)	0.423
Time to first liquid diet	5 (1–22)	4 (1–32)	0.523
Postoperative hospitalization	8 (4–26)	7 (4–55)	0.645
Readmission in 30 days, *n* (%)	2 (4.8)	0 (0)	0.241^a^

a Fisher’s exact test.

Recovery of bowel function was represented by time to first flatus (3 vs 3 days, *P *=* *0.322), defecation (4 vs 4 days, *P *=* *0.423), and liquid diet (5 vs 4 days, *P *=* *0.523), for which no significant difference was observed between the two groups. Postoperative hospital stay was not statistically different between the groups (8 vs 7 days, *P *=* *0.645). There was no readmission in the control group, whereas there were two readmissions in the study group within 30 days: one for anastomotic stenosis and the other for bowel obstruction (*P *=* *0.241).

### Postoperative complications

There were eight and seven patients in the study and control groups, respectively, who developed postoperative complications (19.0% vs 17.1%, *P *=* *0.815). Some patients had more than one complication. In the study group, there were three (7.1%) cases of intra-abdominal infection, one (2.4%) intra-abdominal hemorrhage, two (4.8%) anastomotic leakages, one (2.4%) biliary leakage, one (2.4%) chylous leakage, three (7.1%) duodenal stump leakages, one (2.4%) anastomotic stenosis, and one (2.4%) bowel obstruction. In the control group, there were two (4.9%) intra-abdominal infections, one (2.4%) intra-abdominal hemorrhage, one (2.4%) ascites, one (2.4%) anastomotic leakage, one (2.4%) biliary leakage, one (2.4%) colonic leakage, one (2.4%) duodenal stump leakage, and one (2.4%) gastroplegia. By continuity correction and Fisher’s exact test, all complications were not different between the groups (all *P *>* *0.05, Table 5).

**Table 5.** Postoperative complications

Variables	Study group (*n *=* *42)	Control group (*n *=* *41)	*P-*value
Total	8 (19.0)	7 (17.1)	0.815
Intra-abdominal infection	3 (7.1)	2 (4.9)	1.0^a^
Intra-abdominal hemorrhage	1 (2.4)	1 (2.4)	1.0^b^
Ascites	0 (0)	1 (2.4)	0.494^b^
Anastomotic leakage	2 (4.8)	1 (2.4)	1.0^a^
Biliary leakage	1 (2.4)	1 (2.4)	1.0^b^
Chylous leakage	1 (2.4)	0 (0)	1.0^b^
Colonic leakage	0 (0)	1 (2.4)	0.494^b^
Duodenal stump leakage	3 (7.1)	1 (2.4)	0.626^a^
Gastroplegia	0 (0)	1 (2.4)	0.494^b^
Anastomotic stenosis	1 (2.4)	0 (0)	1.0^b^
Bowel obstruction	1 (2.4)	0 (0)	1.0^b^

a Continuity correction.

b Fisher’s exact test.

## Discussions

### Main findings

When preparing patients for gastric cancer surgery, drinking 250 mL of 5% glucose solution 2–3 hours preoperatively did not increase gastric residual volume, acidity, or perioperative complications. Moreover, it lowered thirst and hunger scores compared with conventional overnight fasting.

### Safety and effectiveness

Pulmonary aspiration is a severe anesthesia-associated complication. If a great amount and acidity of gastric contents reach the bronchial tree through regurgitation, it can cause aspiration pneumonia with a high risk of mortality and severe morbidity [1, 16–18]. Therefore, emptying the stomach through overnight fasting before surgery is believed to help prevent pulmonary aspiration. However, the incidence of pulmonary aspiration was only 1/8,000 in elective ASA status I–II patients [2]. The fatal aspiration rate was even lower, at ∼1/340,000 [19]. A research demonstrated that unrestricted oral fluid ≤3 hours before surgery does not increase gastric residual volume or pH [20]. Another study showed a similar result that ∼95% of liquid was emptied within 2 hours in preoperative patients [21]. Meta-analysis of randomized controlled trials suggested that oral intake of small amounts of non-alcoholic clear fluids 2 hours before surgery would be safe in children and adults undergoing elective procedures [22, 23]. Prolonged fasting has been proven to cause discomfort and stress, and interfere with metabolism, while preoperative intake of carbohydrate-rich drinks could reduce hunger and thirst, and shorten hospital stay [24]. Therefore, shortened fasting time and preoperative oral carbohydrate loading have already been adopted as part of a multidisciplinary, evidence-based guideline for elective colorectal surgery [6]. Although the ERAS Society recommended similar preoperative management for gastrectomy [25], there has always been concern about impaired gastric motility and delayed gastric emptying in gastric cancer patients, not to mention that the evidence used in the guidelines was not specifically based on the study of that population [26–28]. The Japanese Gastric Cancer Treatment Guidelines 2014 (version 4), on the contrary, suggested a clinical pathway for gastrectomy without mentioning reduced preoperative fasting time or preoperative oral carbohydrate supplementation [13]. A recent survey showed that 68.5% of Korean gastric surgeons kept their patients as nil by mouth from midnight and the remaining kept them even longer [29]. Moreover, only ∼10.1% of surgeons provided preoperative carbohydrate drinks to their patients. The lack of convincing direct evidence for fasting and preoperative carbohydrate supplementation in patients with gastric cancer might be the underlying reason that discourages surgeons from changing their conventional habits.

### Gastric residual fluid volume and pH

Previous studies usually used a nasogastric tube with blind aspiration [20, 30, 31] or the dilution method with a radioactive marker to record gastric residual volume [32]. There have always been concerns about the accuracy of both methods, not to mention the radiation issue. In our study, gastroscopy ensured complete suction of gastric residual fluid under direct vision, offering an accurate way to record residue volume. To our knowledge, this was the first study under direct vision to prove that drinking 250 mL of 5% glucose solution 2–3 hours before surgery did not increase the gastric residual volume or lower the pH level of the gastric residual in patients with gastric cancer. No pulmonary aspiration was observed in both groups. Moreover, oral intake of 250 mL of 5% glucose solution significantly eased thirst and hunger in patients with gastric cancer preoperatively, which was concordant with previous research in other types of elective surgery [24, 33]. These results suggested that drinking 250 mL of 5% glucose solution 2–3 hours before surgery in patients with gastric cancer is safe for anesthesia and effective at lowering discomfort for the patients.

### Carbohydrate loading

The stress response to surgery results in increased catabolism and hyperglycemia. Prolonged preoperative fasting exacerbates this response and aggravates insulin resistance after surgery [34, 35]. Preoperative oral carbohydrate loading has been shown to reduce insulin resistance [36]. In our study, we found that 250 mL of 5% glucose solution did not have a significant effect on insulin resistance in gastric cancer patients, as no difference was observed in both the status and the recovery of QUICKI at each time point. Linear mixed model analysis also showed no statistical differences in status or recovery of QUICKI for groups (both *P *<* *0.05) or group×time (both *P *<* *0.05). The laboratory findings representing systemic stress responses such as WBC and CRP were also not different at each time point or in linear mixed models between the groups (all *P *>* *0.05). The reason might be that we used 250 mL of 5% glucose solution instead of 400 mL of carbohydrate-rich (12.5%) solution, which other researchers usually used; therefore, less glucose was taken in, hence the lower effect on metabolism and stress response. Also, more accurate techniques to determine insulin resistance such as hyperinsulinemic–euglycemic clamping could be adopted in future studies. Nevertheless, these results also indicated that oral intake of 250 mL of 5% glucose solution 2–3 hours before surgery alone in gastric cancer patients will not significantly change the stress or metabolism status.

Although a Cochrane Review including 27 trials and 1,976 patients showed that preoperative carbohydrate supplement was associated with a reduction in hospital stay, the variation in study quality and results undermined the actual value of this evidence [37]. Another meta-analysis showed that oral intake of a carbohydrate drink did not reduce complication rates, but data on gastrectomy were inadequate [33]. In the present study, early surgical outcomes, postoperative courses, and postoperative complications were also not statistically different between the two groups.

Li *et al.* showed that, in diabetic patients undergoing gastro-intestinal surgery, preoperative carbohydrate-rich (14.5%) solution loading with individualized supplemental insulin improved postoperative well-being such as thirst, hunger, and fatigue, but did not promote gastro-intestinal recovery [38]. The participants in this study were diabetic patients undergoing gastro-intestinal surgery, and the cases undergoing gastric surgery were only a small portion (18.8% in control group, 12.9% in carbohydrate group). In contrast, we excluded patients with diabetes and enrolled patients with gastric cancer who underwent gastrectomy in our study. Li’s study and our study are complementary, proving the safety and benefits of preoperative carbohydrate supplementation in both non-diabetic and diabetic patients undergoing gastric surgery. The addition of glutamine or whey protein to carbohydrate drinks may increase the benefit [39, 40]. There was also research studying the safety of a protein and carbohydrate combination for preoperative loading. Nogueira *et al.* found that gastric emptying was completed within 3 hours after oral supplement of 200 mL of carbohydrates containing whey protein in healthy young adults [41]. Further study is still needed to investigate the metabolic benefits of a carbohydrate drink containing whey protein as well as its ideal amount and administration time in patients with gastric cancer.

### Limitations

There are several limitations of our study. Firstly, we excluded diabetes patients in our trial. Therefore, research concerning preoperative carbohydrate drinking in diabetes patients is still needed. Secondly, we realized that, in some other developed countries, commercially available 12.5% or higher carbohydrate-rich drinks were adopted preoperatively [11, 32, 42]. Unfortunately, at the time at which our trial was designed and carried out, no similar product was available in most cities in China. In addition, although routine carbohydrate loading is recommended, there is no consensus on the optimal solution or regimen [43], and its clinical benefit is controversial [44, 45]. Considering this was a multicenter study involving hospitals in various areas, the most easily acquired carbohydrate drink in all these hospitals would be a 5% glucose solution. Besides, the lower sweetness of it was also fit for Asian tastes. Thirdly, we used QUICKI as a convenient tool to estimate insulin sensitivity, yet it might not be as accurate as hyperinsulinemic–euglycemic clamping.

## Conclusions

Although there were no obvious improvements in postoperative insulin resistance, recovery of gut function, and hospital stay, drinking 250 mL of 5% glucose solution 2–3 hours before radical gastrectomy in patients with gastric cancer showed benefits in reducing preoperative discomfort such as thirst and hunger, without increasing gastric residual volume, acidity, and perioperative complications. We hope our results can offer direct evidence to help change the traditional clinical practice of overnight fasting in gastric cancer surgery and in turn bring the associated benefits to thousands of gastric cancer patients in the future.

## Supplementary Material

goae077_Supplementary_Data
