# Supplementary figures and images for: Gastric residual volume, safety, and effectiveness of drinking 250 mL of glucose solution 2–3 hours before surgery in gastric cancer patients: a multicenter, single-blind, randomized–controlled trial
Source: Gastroenterol Rep (Oxf). 2024 Sep 12;12:goae077. doi: 10.1093/gastro/goae077 (PMC11398872; doi:10.1093/gastro/goae077)

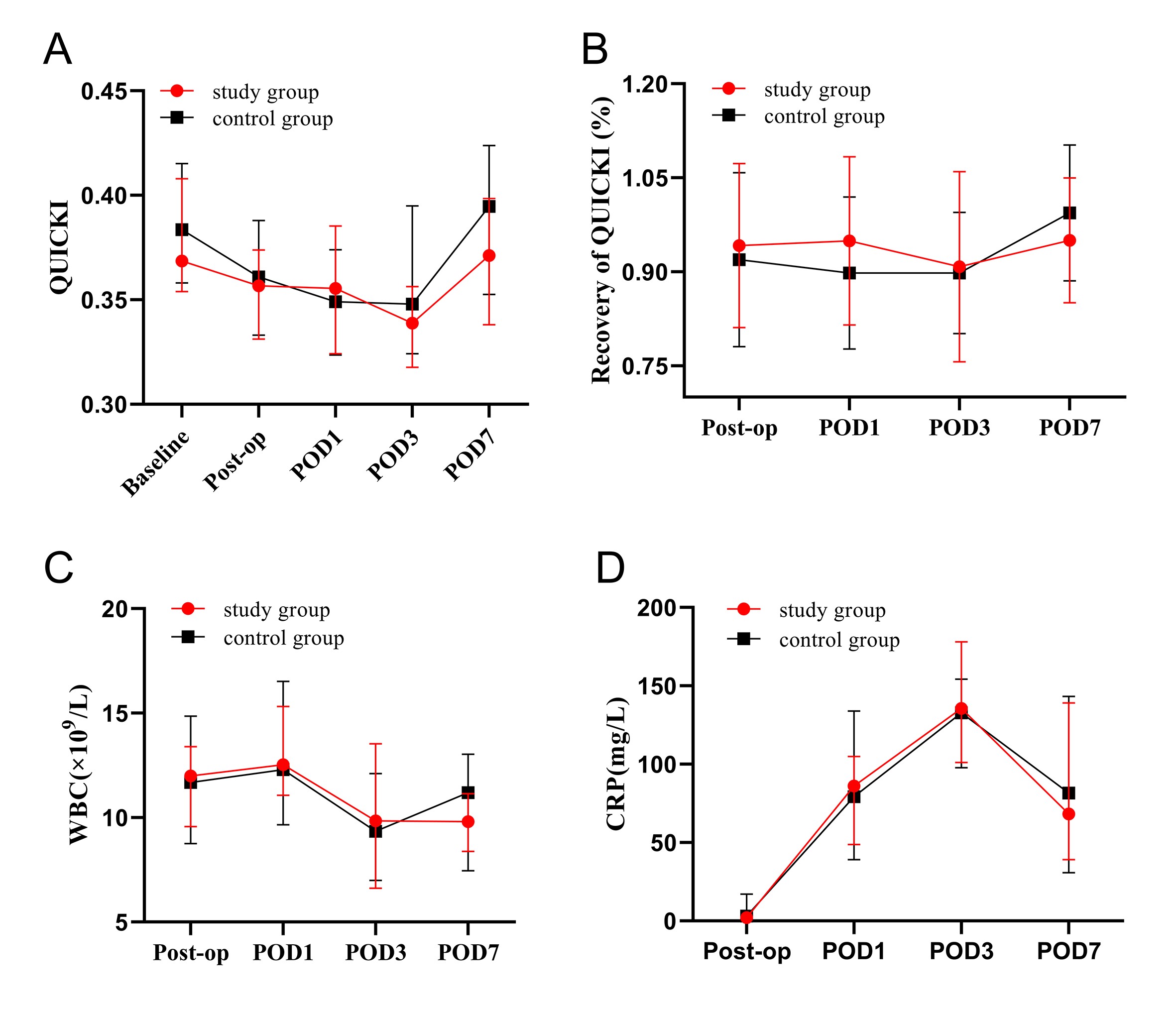

Supplement: goae077_Supplementary_Data [file goae077_supplementary_data.jpeg]
